# Soil Bacterial Diversity Responds to Long-Term Establishment of Perennial Legumes in Warm-Season Grassland at Two Soil Depths

**DOI:** 10.3390/microorganisms11123002

**Published:** 2023-12-18

**Authors:** Adesuwa Sylvia Erhunmwunse, Victor Alonso Guerra, Jung-Chen Liu, Cheryl L. Mackowiak, Ann Rachel Soffes Blount, José Carlos Batista Dubeux, Hui-Ling Liao

**Affiliations:** 1 North Florida Research and Education Center, University of Florida, 155 Research Road, Quincy, FL 32351, USAsunny.liao@ufl.edu (H.-L.L.); 2 Soil, Water and Ecosystem Sciences Department, University of Florida, Gainesville, FL 32611, USA; 3 North Florida Research and Education Center, University of Florida, 3925 Highway 71, Marianna, FL 32446, USA; dubeux@ufl.edu

**Keywords:** rhizoma peanut, bahiagrass, alpha diversity, beta diversity, N fixing bacteria

## Abstract

The introduction of rhizoma peanut (RP *Arachis glabrata* Benth) into bahiagrass (*Paspalum notatum* Flüggé) may require time to develop stable plant–soil microbe interactions as the microbial legacy of the previous plant community may be long-lasting. A previous study showed that <2 years of introducing rhizoma peanut into bahiagrass pastures minimally affected soil bacterial diversity and community composition. In this study, we compared the effects of the long-term inclusion of rhizoma peanut (>8 years) into bahiagrass on soil bacterial diversity and community composition against their monocultures at 0 to 15 and 15 to 30 cm soil depths using next-generation sequencing to target bacterial 16S V3–V4 regions. We observed that a well-established RP–bahiagrass mixed stand led to a 36% increase in bacterial alpha diversity compared to the bahiagrass monoculture. There was a shift from a soil bacterial community dominated by Proteobacteria (~26%) reported in other bahiagrass and rhizoma peanut studies to a soil bacterial community dominated by Firmicutes (39%) in our study. The relative abundance of the bacterial genus *Crossiella*, known for its antimicrobial traits, was enhanced in the presence of RP. Differences in soil bacterial diversity and community composition were substantial between 0 to 15 and 15 to 30 cm soil layers, with N_2_-fixing bacteria belonging to the phylum Proteobacteria concentrated in 0 to 15 cm. Introducing RP into bahiagrass pastures is a highly sustainable alternative to mineral N fertilizer inputs. Our results provide evidence that this system also promotes greater soil microbial diversity and is associated with unique taxa that require further study to better understand their contributions to healthy pastures.

## 1. Introduction

The integration of legumes into grasslands has gained renewed attention due to their potential to lower nitrogen (N) fertilizer inputs, improve soil health, enhance nutrient cycling, and minimize environmental damage associated with the excessive use of inorganic N fertilizer [1,2,3]. Rhizoma peanut (*Arachis glabrata* Benth.; RP) is a tropical perennial legume well adapted to pasture systems in the southeastern United States. It persists under grazing [4]. Extensive research has been conducted on the agronomic and livestock benefits of introducing RP into bahiagrass (*Paspalum notatum* Flüggé) pastures, a major warm-season perennial grass also adapted to the southeastern United States [4,5,6,7,8]. When compared to bahiagrass receiving N fertilizer, RP inclusion into a pasture elevates forage nutritive value and animal performance [5,6,7].

Likewise, desirable soil microbial associations with RP have also been observed [9,10,11,12]. For example, the presence of RP in bahiagrass systems was associated with increases in soil microbial diversity and the relative abundance of soil microbes with potential N_2_-fixing and cycling abilities, such as *Bradyrhizobium* and members of the *Allorhizobium*-*Neorhizobium*-*Pararhizobium*-*Rhizobium* clade [10,11]. Plants influence soil microbial communities mainly through rhizodeposition and plant litter accumulation [13]. The impacts of plants on soil microbes are dynamic, with outcomes that are likely to change as plant growth and development progresses [14,15]. Kulmatiski and Beard [16] demonstrated that plants require several years to assemble soil microbial communities and that the development of plant–soil–microbe feedback should be viewed in a longer-term context in perennial plant systems. Hannula et al. [17] reported that soil microbiomes are reversible and plant–soil–microbe feedback characteristics at an early plant development stage can change with time. In a recent study, we observed that the bacterial alpha and beta diversities and the relative abundance of individual taxa within the first two years of RP integration into an existing bahiagrass system were not different from those in bahiagrass monoculture [12]. Rhizoma peanut establishment is achieved through the vegetative propagation of rhizomes and requires two to three years for stands to fully establish [18,19]. As a result, this longer establishment period likely delays RP impacts on soil microbial communities. 

Many microbial studies on RP have targeted the upper 10 to 15 cm soil depth [9,10,11,12]. The surface soil likely harbors greater microbial density because of greater root densities and plant litter accumulation, which also results in greater soil carbon [20,21,22]. Microbial diversity has been linked to the sustainability and productivity of grasslands by enhancing ecosystem services such as nutrient cycling [23,24]. Microbial diversity and community composition have differed between 0 to 15 and 15 to 30 cm, showing the differentiation of these microbial communities between these layers [20,22]. In this study, we compared the bacterial diversity and community composition in bahiagrass–RP mixtures versus their monocultures at two soil depths. We hypothesized that compared to bahiagrass monoculture, a well-established bahiagrass–RP mixed stand will result in greater bacterial diversity and increase the relative abundance of key soil bacterial taxa, especially near the surface soil (0 to 15 cm depth). Our objectives were to determine the longer-term (>8 years) impact of RP introduction into bahiagrass on soil bacterial diversity and community composition at two soil depths (0 to 15 and 15 to 30 cm). Overall, our results will provide valuable insights into the bacterial groups and the potential functions promoted by RP establishment in bahiagrass stands.

## 2. Materials and Methods

### 2.1. Experimental Site

The study was conducted in 2020 at the University of Florida, North Florida Research and Education Center, Quincy, Florida (30°33′ N, 84°36′ W). The soil was classified as Orangeburg loamy sand series (fine-loamy, kaolinitic, thermic Typic Kandiudults) [25]. The average temperature in 2020 was 20.0 °C (6.7 and 31.7 °C min/max, respectively). Annual rainfall was 1781 mm in 2020 compared to the 30-year average of 1519 mm [26]. The experimental site was initiated in 2011 and consisted of eight treatments, including two bahiagrass entries, two RP entries, and their mixtures, arranged in a randomized complete block design with three replicates. Detailed information on site establishment and management through 2017 is reported by Santos et al. [6] and Santos et al. [27]. In this study, only three of the existing forage treatments, Ecoturf RP (Eco), Argentine bahiagrass (Arg), and their binary mixtures (Arg-Eco), were sampled. Each plot measured 9 m^2^ with 1.2 m-wide alleys between plots. The average soil fertility of the soil samples collected from each plot was measured as follows for 0–15 cm and 15–30 soil depths: pH (2:1 water: soil) = 5.9 and 5.8, total soil carbon = 12 and 6 g kg^−1^, CEC = 4.6 and 3.4 cmol^+^ kg^−1^, Mehlich-3 extractable P, K, and Mg of 28 and 16, 76 and 49, and 79 and 44 mg kg^−1^. In 2019, there was a rotary cut in each plot at the end of the growing season. In March 2020, the RP monoculture plots were staged in March and treated for winter weeds in April 2020. Imazapic was applied at a rate of 291 mL ha^−1^ (0.07 kg a.i. ha^−1^), and Clethodim was applied ten days later at a rate of 877 mL ha^−1^ (0.21 kg a.i. ha^−1^). The grass monoculture and binary mixture treatments were mowed but not treated with herbicides. 

### 2.2. Soil Sampling and Analysis

Soil samples (combined bulk and rhizosphere) were collected in July 2020 using a hydraulic soil coring probe (Giddings Machine Company, Windsor, CO, USA). Four soil cores (4.4 cm dia.) were collected from two soil depths (0 to 15 and 15 to 30 cm) per plot and composited. Subsamples were placed in 15 mL centrifuge tubes, frozen at −80 °C for 48 h, freeze-dried for 24 h and stored at −80 °C until DNA extraction. Genomic DNA was extracted from soil samples following the DNeasy PowerSoil extraction kit instructions (Qiagen, Hilden, Germany). DNA quantity and quality were measured using NanoDropTM One (Thermo Scientific, USA) based on the absorbance ratios (A260/A280 and A260/A230). Bacterial 16S rRNA gene V3–V4 regions were amplified using primer pairs 341F/806R [28]. Amplicon libraries were prepared using two PCR amplification steps described by Chen et al. [29] and using a Labnet MultiGene Optimax Thermal Cycler (Labnet International Inc., Edison, NJ, USA). At the second PCR stage, barcode tags unique to individual samples were tagged to the tail of the reverse primer. PCR products from the first and second steps were purified using AMPure XP beads (Beckman Coulter, Inc., Indianapolis, IN, USA) following the manufacturer’s instructions and observed on 1% agarose gels. Generated amplicon libraries were pooled at 20 ng/µL and sent to Duke Center for Genomic and Computational Biology (GCB) for Illumina MiSeq sequencing (v3 300 bp, 13 Gb) and paired-end reads were generated.

### 2.3. Bioinformatic and Statistical Analysis 

The prokaryotic 16S rRNA gene sequences were processed in QIIME2 [30]. Primers were removed using cutadapt version 3.4 in QIIME2 version 2021.4 [30]. DADA2 was used for quality filtering and removal of chimeric sequences. Sequences at 100% similarity were assigned to the same amplicon sequence variant (ASV). Silva 138.1 database (version 2020) was used to assign taxonomic information. Singletons, unassigned, and reads that were classified as mitochondria and chloroplast were removed.

The ASV table (100% sequence similarity) was rarefied at 5000 sequencing depth and used for alpha and beta diversity. Alpha diversity indices were measured in QIIME2 using the Shannon index and observed features and analyzed using linear mixed model in R version 4.2.1 [31]. Forage treatment and soil depths were considered fixed effects and block was used as the random effect. When the main effect of forage treatment was significant, pairwise comparisons were carried out using Tukey’s post hoc test. In addition to graphical observations, Shapiro–Wilk’s test was used to assess the normal distribution of the residuals of our variables, and Bartlett’s test was used to determine the equality of variance. Beta diversity was estimated using the Bray–Curtis dissimilarity-based principal coordinates analysis with the vegdist function in vegan package. A test for homogeneity of multivariate dispersions (PERMDISP) within treatments was conducted [32] using 9999 permutations. PERMDISP was performed using the betadisper function in the vegan version 2.6-4 [33], and homogenous dispersion was observed within treatments. Dissimilarities of soil bacterial community composition in response to forage treatments, soil depth, and their interaction were tested using nonparametric permutational multivariate ANOVA [34] using 9999 permutations with the Adonis function in the vegan package. Pairwise comparisons were made for significant main effect of forage treatment using ADONIS. 

## 3. Results

### 3.1. Soil Bacterial Community Composition

**Figure 1:**
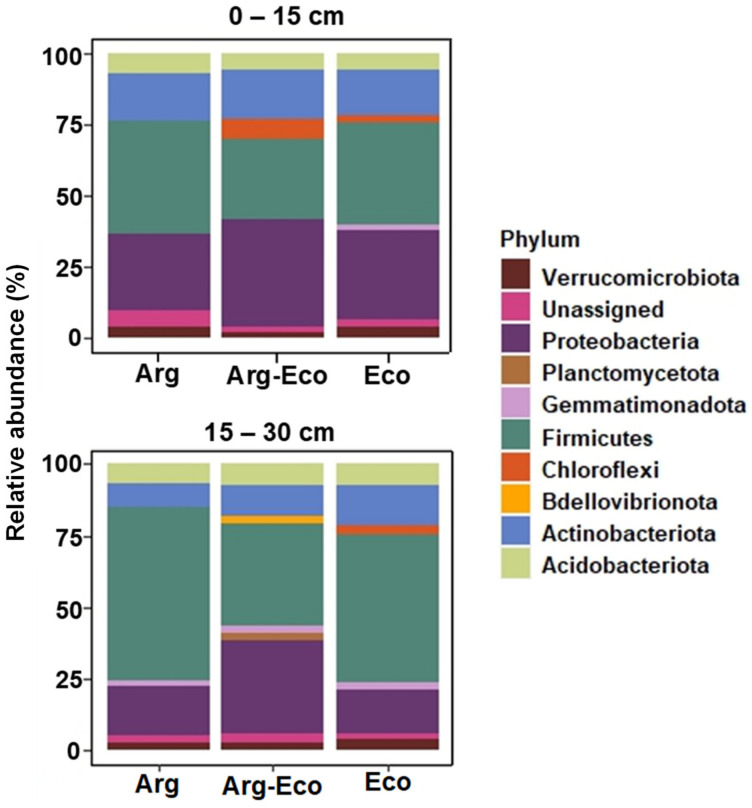
Relative abundance (>1%) of major bacterial phyla under three forage treatments (Arg, Arg-Eco, and Eco) at two soil depths (0 to 15 and 15 to 30 cm).

Across all forage treatments and soil depths, dominant phyla included Firmicutes (39 ± 4.3%), Proteobacteria (25 ± 2.3%), Actinobacteriota (15 ± 1.5%), and Acidobacteriota (7 ± 0.6%). Phyla representing <5% relative abundance included Chloroflexi (3.1 ± 0.7%), Verrucomicrobiota (2.9 ± 0.3%), Planctomycetota (2.0 ± 0.3%), and Gemmatimonadota (1.4 ± 0.2%), and Myxococcota (1.0 ± 0.1) (Figure 1). Unassigned and rare (<0.5%) phyla accounted for 1.8 ± 1.1% of all sequences. Approximately 76% of total sequences were assigned at the genus level. *Bacillus* (27.1 ± 3.0; Firmicutes), *Rhodoplanes* (8.9 ± 1.0; Proteobacteria), *Tumebacillus* (3.2 ± 0.8; Firmicutes), *Paenibacillus* (3.1 ± 0.7; Firmicutes), and *Massilia* (3.0 ± 0.7; Proteobacteria) were among the major bacterial genera, with *Bacillus* and *Rhodoplanes* accounting for 36% of the total bacterial genera.

### 3.2. Soil Bacterial Diversity in Response to Forage Treatment and Soil Depth

There were no significant two-way interactions between forage treatment and soil depth on bacterial alpha diversity measured in terms of the observed features or Shannon diversity index, but forage treatment and soil depth significantly (*p* < 0.05) affected bacterial alpha diversity (Table 1). Among all forage treatments, Ecoturf RP (Eco) and Arg-Eco mixture exhibited greater bacterial alpha diversity than bahiagrass monoculture (Arg). Observed features and Shannon diversity were 20 and 6%, respectively, greater in 0 to 15 cm than in 15 to 30 cm. 

Bacterial beta diversity showed that soil bacterial community composition was significantly (*p* < 0.05) affected by forage treatment, soil depth, and their interaction (Table 2; Figure S1). At the 0 to 15 cm soil depth, soil bacterial community composition was distinct within each forage treatment. However, at 15 to 30 cm soil depth, soil bacterial community composition in Eco was dissimilar from those in the mixture (Arg-Eco) and bahiagrass monoculture (Arg) (Figure S1). Both Arg and Arg-Eco plots had overlapping soil bacterial communities. Soil depth explained 28% of the variation in soil bacterial community composition compared to 14% explained by forage treatment (Table 2).

### 3.3. Core Microbiome and Soil Bacterial Biomarkers 

Core microbiomes were identified based on the presence of the ASVs in 50% of the samples across forage treatments and soil depths. Amplicon sequence variants identified at genus level included *Bacillus* (Firmicutes, 27.1 ± 2.9%), *Rhodoplanes* (Proteobacteria, 8.9 ± 1.0%), *Tumebacillus* (Firmicutes, 3.3 ± 0.8%), *Bradyrhizobium* (Proteobacteria, 2.2 ± 0.3%), *Candidatus* Udaeobacter (Verrucomicrobiota, 2.3 ± 1.0%), and *Streptomyces* (Actinobacteriota, 2.1 ± 0.8%) (Figure S2). Effects of forage treatment, soil depth, and their interaction were determined on the relative abundances of the core microbiome (Table S1). *Crossiella* and *Phenylobacterium* were influenced by forage treatment (Figure S3A). *Crossiella* was greater in Eco than Arg and Arg-Eco, while *Phenylobacterium* was enhanced in plots containing RP (Eco and Arg-Eco). *Bradyrhizobium*, *Crossiella*, *Lysinibacillus*, *Paenibacillus*, *Rhodanobacter*, *Sphingomonas*, and Subgroup_2 (Phylum: Actinobacteria) were affected by soil depth (Table S1). *Bradyrhizobium*, *Lysinibacillus*, and *Sphingomonas* were enriched in 0 to 15 cm soil depth and others were dominant in 15 to 30 cm soil depth (Figure S3B). Only *Tumebacillus* was affected by the interaction of forage treatment and soil depth (Table S1), where Eco demonstrated a greater relative abundance than the mixture, followed by Arg (Figure S3C).

**Figure 2:**
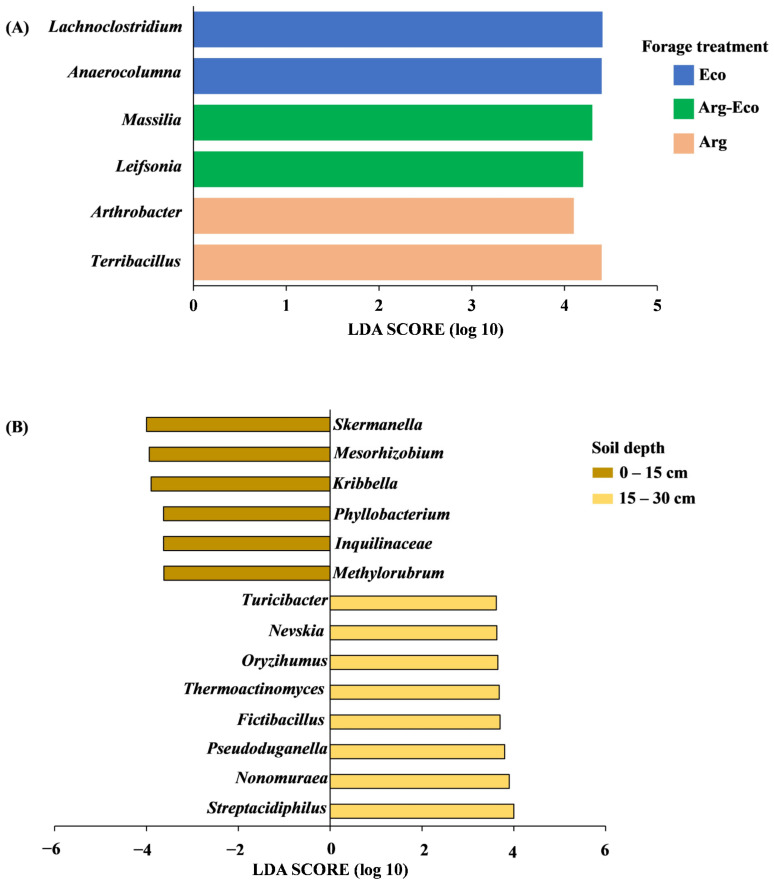
Differential abundant bacterial genera among (**A**) forage treatments and (**B**) soil depths based on Lefse analysis. Differences in abundance among forage species (**A**) and soil depth (**B**) are coded by color (see legends). The linear discriminant analysis effect size (LEfSe) of microbial communities with LDA scores greater than 2.0 was considered.

Lefse analysis detected two abundant bacterial genera within each forage treatment, with *Lachnoclostridium* and *Anaerocolumna* (Firmicutes) identified in Eco, *Massilia* (Proteobacteria) and *Leifsonia* (Actinobacteriota) in Arg-Eco, and *Arthrobacter* (Actinobacteriota) and *Terribacillus* (Firmicutes) in Arg (Figure 2A). Fourteen bacterial genera were identified across soil depths, with six in 0 to 15 cm and eight in 15 to 30 cm (Figure 2B). Apart from *Kribbella* (Actinobacteriota), bacterial genera belonging to Proteobacteria were mainly present at the 0 to 15 cm soil depth and included *Skermanella*, *Mesorhizobium*, *Phyllobacterium*, *Inquilinaceae*, and *Methylorubrum*. Bacterial genera identified at 15 to 30 cm soil depth were a mixture of phyla Actinobacteriota (*Nonomuraea*, *Oryzihumus*, and *Streptacidiphilus*), Firmicutes (*Fictibacillus*, *Thermoactinomyces*, and *Turicibacter*), and Proteobacteria (*Nevskia* and *Pseudoduganella*). 

## 4. Discussion

### 4.1. Soil Bacterial Diversity and Community Response 

Legumes incorporated into grasslands often enhance soil bacterial diversity and community composition directly through the supply of N from biological N_2_ fixation and the release of N-rich root exudates and plant litter [13,35,36]. Increased microbial diversity in agroecosystems has been linked to increases in ecosystem services, such as nutrient cycling and nutrient availability [24,37], as well as resilience to environmental disturbances [38,39]. In support of our hypothesis, the long-term (>8 years) incorporation of RP into a bahiagrass system enhanced soil bacterial diversity and the relative abundance of core bacterial taxa. This result is in contrast to a previous study [12], where <2 years of RP establishment in an existing bahiagrass system did not alter soil microbial diversity relative to the bahiagrass monoculture. Prior existing plant communities can have a strong effect on soil community composition. However, the association between introduced plant species and soil bacteria may change over time within the same ecosystem as plant growth and development advances [40,41]. Schmid et al. [15] showed that the effect of old/existing grassland communities on soil microbial communities prior to the reassembling of new plant communities may still be identifiable after 4 years. The authors elaborated that the impact of a plant community on soil communities is a gradual process and requires more time to fully develop. In comparison, Hannula et al. [17] discovered that the influence of prior existing Poaceae and Asteraceae species on soil bacterial communities faded quickly with the introduction of new Poaceae and Asteraceae species. 

Ecoturf RP and its mixture with bahiagrass had greater bacterial alpha diversity than bahiagrass monoculture. Factors such as soil properties and environmental conditions can drive microbial community structure in agricultural systems. However, plant species with divergent above and belowground properties also can exert a strong selective effect on soil bacterial communities [13,35]. For example, a concurrent study showed that RP root + rhizomes biomass and N concentrations were greater than bahiagrass root + rhizomes [42]. Furthermore, we found a 71% increase in rhizoma peanut root + rhizome biomass from 2015/2016 [43] to 2020 [42] on the same study site with greater annual rainfall in 2016 and similar temperature across study years. Often, perennial plants tend to accumulate belowground biomass over several years.

Interestingly, the bacterial community shifted from being dominated by Proteobacteria, as reported in other RP studies [10,11,12,44], to Firmicutes in our study. Reasons for the shift are not well understood, but Firmicutes have been reported in older, stable agroecosystems [45]. Different plant species have been linked to unique microbial taxa due to differences in plant litter quality and root exudate composition [36,44]. Guerra et al. [10] showed that *Bryobacter*, which is associated more with abundant C substrates, dominated a bahiagrass system, while RP had a greater relative abundance of *Crossiella*. Similarly, in our study, the relative abundance of *Crossiella* was enhanced under Ecoturf RP plots. *Crossiella* has been documented to have antimicrobial properties that can inhibit soil-borne pathogens [46]. *Crossiella* might provide an indirect defense mechanism against pathogens [46,47]. In our study, we observed that bacterial taxa capable of metabolizing a wide range of plant polysaccharides were found in bahiagrass and Ecoturf RP plots. For example, in Ecoturf RP plots, anaerobic, spore-forming bacteria, *Anaerocolumna* and *Lachnoclostridium* were identified. These require amino acids/peptides as a source of N for the decomposition of plant materials [48,49]. In comparison, the bahiagrass plots contained non-spore-forming and siderophore-producing *Arthrobacter* and aerobic *Terribacillus*, [50,51] known to utilize different organic compounds and thought to be involved in the transformation of soil organic matter [52].

### 4.2. Soil Depth Influences Variation in Soil Microbial Communities

Soil depth had a noticeable influence in shaping soil bacterial communities, as evident in the 28% variation of soil bacterial communities explained by soil depth. The impact of soil depth on soil bacterial communities has been attributed to changes in plant root systems and soil physicochemical properties, including soil C, soil moisture, and soil temperature, within soil depths [22,53]. Soil bacterial alpha diversity between 0 to 15 and 15 to 30 cm soil depths showed that 0 to 15 cm had greater bacterial diversity. It is well-established that microbial diversity is greater in the surface soil due to greater accumulation of organic matter inputs [20,21,22]. Guerra [42] reported that most of the total root length and root mass density observed for Argentine bahiagrass and Ecoturf RP was in the surface 0 to 15 cm. Rhizodeposits and root and rhizome litter provide soil carbon and nutrient resources for soil microbes, thereby likely creating niche differentiation for soil bacteria [20,53].

We found that members of the phylum Proteobacteria were enriched in the 0 to 15 cm. Interestingly, most of the bacterial genera were potential N_2_ fixers, including members of *Bradyrhizobium*, *Skermanella*, and *Mesorhizobium*. The concentration of N_2_-fixing bacteria in the surface soil, compared to 15 to 30 cm depth, might be related to a greater presence of roots/nodules and soil C [54,55]. Bacterial genera differentially selected at the 15 to 30 cm soil depth were a mixture of phyla with diverse putative functions, such as N_2_ fixation and P solubilization (*Paenibacillus*; Firmicutes) [56], denitrification (*Rhodanobacter*; Proteobacteria) [57], biodegradation (*Fictibacillus*; Firmicutes) [58], and release of plant beneficial metabolites (*Nonomuraea*; Actinobacteriota) [59]. This diversity in bacterial genera and their functions highlights the complex and dynamic interactions within bahiagrass–RP mixture, emphasizing the need for further research to unravel soil bacteria roles and contributions in pasture production.

## 5. Conclusions

In this study, we found increases in soil bacterial diversity and shifts in community composition in response to the long-term integration of RP into the bahiagrass system. There was a shift in previously reported Proteobacteria-dominated early establishment systems to a Firmicute-dominated system in our study. Substantial microbial differentiation was observed between 0 to 15 and 15 to 30 cm, with N_2_-fixing bacteria belonging to the phylum Proteobacteria dominating surface soil (0 to 15 cm depth). A mixture of bacterial phyla with diverse plant-promoting abilities was enriched in the 15 to 30 cm soil. Well-established (several years) RP in bahiagrass pastures increases soil bacterial diversity and enhance soil microbial taxa that have the potential to improve plant litter decomposition and suppress plant pathogens present in soil. However, further study is needed to better understand the roles of these soil bacterial taxa in maintaining efficient and healthy pastures. Our findings demonstrate the importance establishment time for perennial forages has in shaping soil microbial community composition. This study, however, only reports data from one time-point; there is a need to conduct more studies across different time points and to consider more long-term studies to understand the interactions within soil microbial communities in perennial forage systems.

## Figures and Tables

**Table 1:** Bacterial alpha diversity indices (observed features and Shannon diversity) under three forage treatments and two soil depths.

	Observed Features	Shannon Diversity
Forage treatment (FT)		
Arg	353 c	7.3 c
Arg-Eco	481 b	7.8 b
Eco	508 a	8.1 a
Soil depth (D)		
0 to 15	486 A	8.0 A
15 to 30	408 B	7.5 B
Source of variation	*p* values ^1^	
FT	0.024 *	0.046 *
D	0.047 *	0.049 *
FT × D	0.439	0.643

^1^ *p* values from ANOVA table showing the effect of forage treatment, soil depth, and their interactions on bacteria alpha diversity. * indicate significance at *p* < 0.05. Pairwise comparisons were conducted using Tukey’s analysis. Lowercase letters show significant differences among forage treatments and uppercase letters show significant differences between soil depths.

**Table 2:** PERMANOVA partitioning and analysis of forage treatment (FT), soil depth (D), and their interactions (FT × D) based on Bray–Curtis dissimilarities of amplicon sequence variant counts.

Source of Variation	Df	MS	Pseudo *F*	R^2^	*p*
FT	2	0.271	1.822	0.14	0.024 *
D	1	1.097	7.362	0.28	<0.001 ***
FT × D	2	0.244	1.402	0.12	0.047 *

* and *** show significance at *p* < 0.05 and <0.0001, respectively.

## Data Availability

The raw reads used in this study can be found at online repository (http://trace.ncbi.nlm.nih.gov/Traces/sra/ under bioproject accession number, PRJNA835799, with submission ID for bacteria (SUB13901473).

## Supplementary Materials

The following supporting information can be downloaded at: https://www.mdpi.com/article/10.3390/microorganisms11123002/s1, Figure S1: Principle coordinates analysis; Figure S2: A heatmap showing core microbiome; Figure S3: The effect of (A) forage treatment, (B) soil depth, and (C) forage treatment x soil depth on the relative abundance of significant core microbiome; Table S1: ANOVA table showing the effect of forage treatment (FT), soil depth (D), and their interactions (FT × D) on the relative abundance of core soil microbiome at the genus level.
