# Supplementary material for: Soil Bacterial Diversity Responds to Long-Term Establishment of Perennial Legumes in Warm-Season Grassland at Two Soil Depths
Source: Microorganisms. 2023 Dec 18;11(12):3002. doi: 10.3390/microorganisms11123002 (PMC10745480; doi:10.3390/microorganisms11123002)
Supplement: Supplementary file 1 [file microorganisms-11-03002-s001.zip › microorganisms-2720192-supplementary.pdf]

Supplementary Data

Soil bacterial diversity responds to long-term establishment of perennial legumes in warm-season grassland at two soil depths

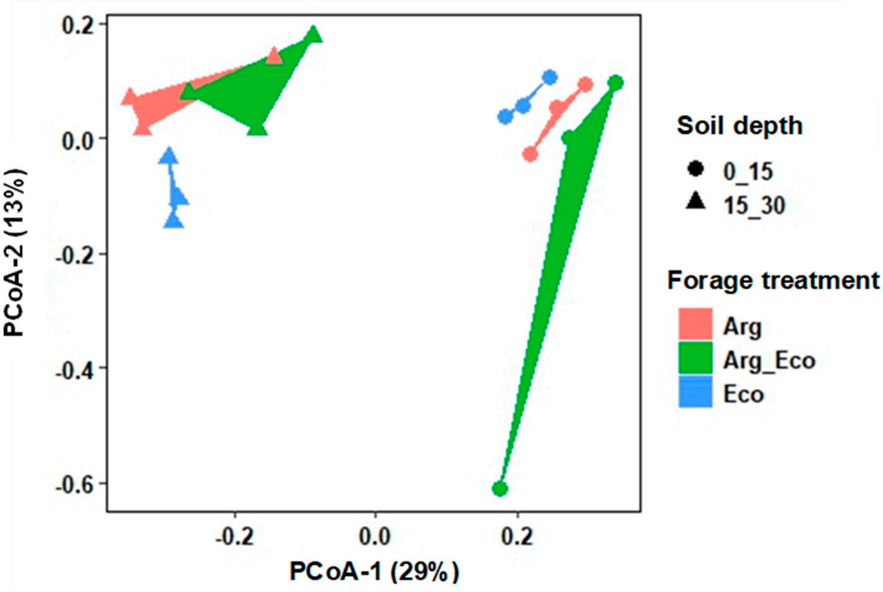

Figure S1. Soil bacterial beta diversity under different forage treatments at two soil depths (0-15 and 15-30 cm). Principle coordinates analysis (PCoA) of Bray–Curtis dissimilarities calculated using amplicon sequence variant counts.

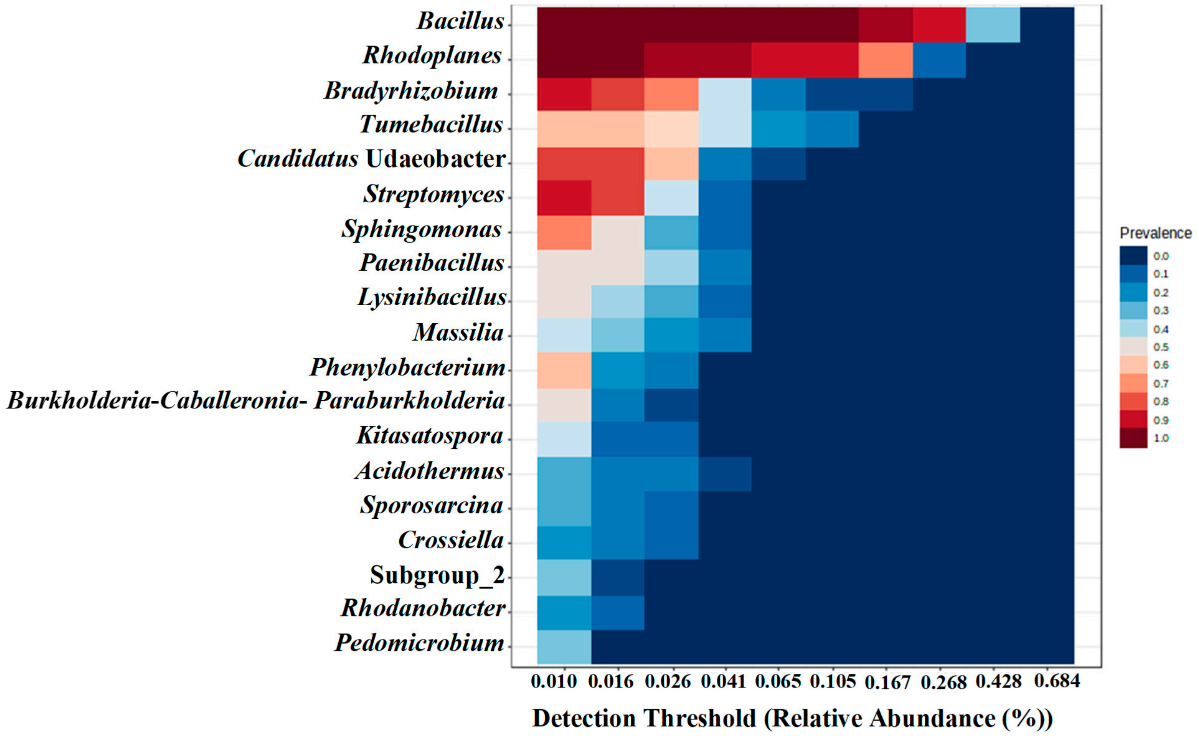

Figure S2. A heatmap showing core microbiome (identified at genus level) across all samples. Blue color indicates that the genus is either absent or present in low abundance, whereas the red color signifies that it is highly abundant.

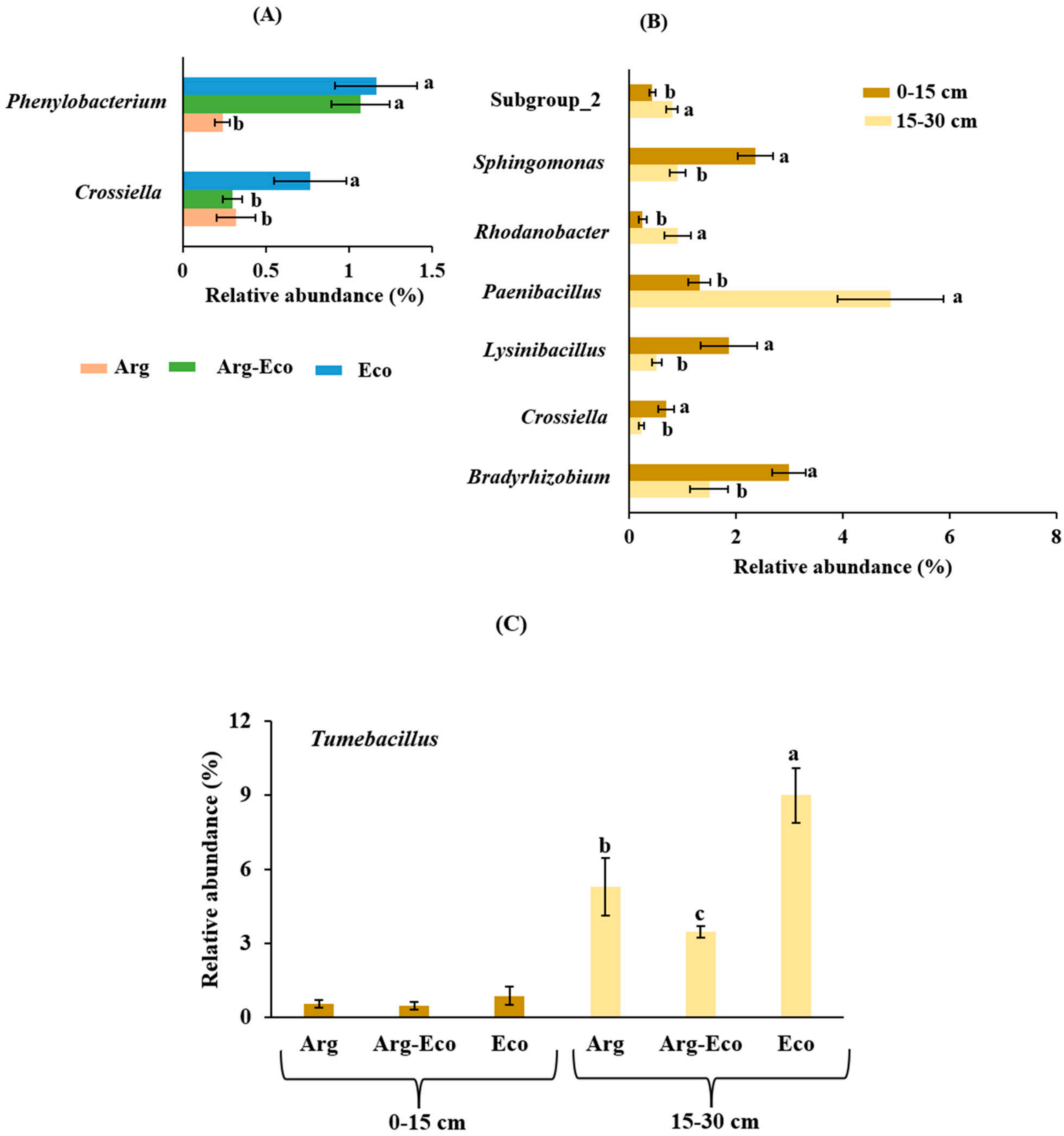

Figure S3. The effect of A) forage treatment, B) soil depth, and C) forage treatment x soil depth on the relative abundance of significant core microbiome based on the P values from ANOVA on table 3. Lowercase letters show significant differences among forage treatments and between soil depths for figure A and B. For figure C, lowercase and uppercase letters show significant differences among forage treatments at 0- 15 and 15- 30 cm, respectively.

Table S1. *P* values from ANOVA table showing the effect of forage treatment (FT), soil depth (D), and their interactions (FT x D) on the relative abundance of core soil microbiome at the genus level.

Bacterial genera	FT	D	FT x D
Acidothermus	0.512	0.232	0.615
Bacillus	0.176	0.182	0.770
Bradyrhizobium	0.114	0.035*	0.531
Burkholderia-Caballeronia-Paraburkholderia	0.535	0.136	0.214
Candidatus Udaeobacter	0.825	0.683	0.283
Crossiella	0.011*	0.003**	0.140
Kitasatospora	0.963	0.747	0.209
Lysinibacillus	0.830	0.048*	0.608
Massilia	0.762	0.176	0.281
Paenibacillus	0.080	0.008**	0.340
Pedomicrobium	0.583	0.783	0.123
Phenylobacterium	0.009**	0.648	0.804
Rhodanobacter	0.650	0.029*	0.384
Rhodoplanes	0.389	0.053	0.387
Sphingomonas	0.131	0.003**	0.473
Sporosarcina	0.431	0.094	0.150
Streptomyces	0.576	0.177	0.051
Subgroup_2	0.395	0.025*	0.851
Tumebacillus	0.003**	0.0001***	0.008**

*, **, and *** indicate significance at $P< 0.05$, 0.01 and<.0001, respectively.
